# Reduced Dendritic Cells Expressing CD200R1 in Children with Inflammatory Bowel Disease: Correlation with Th17 and Regulatory T Cells

**DOI:** 10.3390/ijms161226143

**Published:** 2015-12-04

**Authors:** Mohamed F. Elshal, Alia M. Aldahlawi, Omar I. Saadah, J. Philip McCoy

**Affiliations:** 1 Biochemistry Department, Faculty of Sciences, King Abdulaziz University, Jeddah 21589, Saudi Arabia; 2 Inflammatory Bowel Disease Research Group, King Abdulaziz University, Jeddah 21589, Saudi Arabia; alia008@hotmail.com (A.M.A.); osaadah@kau.edu.sa (O.I.S.); mccoyj@nhlbi.nih.gov (J.P.M.); 3 Molecular Biology Department, Genetic Engineering and Biotechnology Research Institute, Sadat City University, Sadat City 32897, Egypt; 4 Biological Sciences Department, Faculty of Sciences, King Abdulaziz University, Jeddah 21589, Saudi Arabia; 5 Immunology Unit, King Fahd Medical Research Center, King Abdulaziz University, Jeddah 21589, Saudi Arabia; 6 Department of Pediatrics, Faculty of Medicine, King Abdulaziz University, Jeddah 21589, Saudi Arabia; 7 Flow Cytometry Core Facility, National Heart, Lung, and Blood Institute, National Institutes of Health, Bethesda, MD 20892, USA

**Keywords:** inflammatory bowel disease, ulcerative colitis, Crohn’s disease, plasmacytoid dendritic cells, myeloid dendritic cells, CD200R1, CD200, TH17 and regulatory T cells

## Abstract

Loss of tolerance of the adaptive immune system towards indigenous flora contributes to the development of inflammatory bowel diseases (IBD). Defects in dendritic cell (DC)-mediated innate and adoptive immune responses are conceivable. The aim of this study was to investigate the expression of the inhibitory molecules CD200R1 and their ligand CD200 on DCs, to clarify the role of the DCs in the pathogenesis of IBD. Thirty-seven pediatric IBD patients (23 with Crohn’s disease (CD) and 14 with ulcerative colitis (UC)) with mean age 13.25 ± 2.9 years were included. Fourteen age-matched healthy pediatric volunteers (five males and nine females) served as a control group (HC). The percentage of CD11c^+^ myeloid dendritic cells (mDCs) and CD123^+^ plasmacytoid DCs (pDCs) expressing CD200R1 and CD200 were evaluated in peripheral blood using flow cytometry and were correlated with routine biochemical, serological markers, serum levels of cytokines and with the percentages of circulating regulatory T cells (Treg) and CD4^+^ producing IL-17 (Th17). IBD patients showed a significant decrease in the percentage of pDCs and mDCs expressing CD200R1 compared to that of HC. Patients with UC showed increased expressions of the CD200 molecule on pDCs as compared to HC. DCs expressing CD200R1 were found to be correlated positively with Treg and negatively with TH17 and erythrocyte sedimentation rate (ESR). Our findings suggest that IBD is associated with dysregulation in the CD200R1/CD200 axis and that the decrease in DCs expressing CD200R1 may contribute to the imbalance of Th17 and Treg cells and in the pathogenesis of IBD.

## 1. Introduction

Inflammatory bowel diseases (IBD) are chronic autoimmune diseases that affect the small bowel and/or the colon and are comprised mainly of Crohn’s disease (CD) and ulcerative colitis (UC) [1]. Recent epidemiological studies suggest an increase in the incidence of pediatric IBD, with about 10% of cases with IBD occurring in children less than 10 years old [2,3,4]. Several risk factors have been associated with these epidemiologic changes, including excessive use of antibiotics [5], shifts towards a more Westernized high fat, a high carbohydrate diet and urbanization [6]. It has been suggested that these factors alter the composition of the intestinal microflora and induce exaggerated inflammatory responses to these alterations in genetically-susceptible hosts [7,8]. These bacterial changes may result in the disturbance of immune tolerance and cause immune alterations, such as secreting inflammatory mediators, recruiting dendritic cells (DCs) and presenting antigens to T helper (CD4^+^) lymphocytes [9,10].

DCs are a special subset of antigen-presenting cells (APCs) that are widely distributed in all peripheral tissues, including the intestinal mucosa. In the presence of infection or inflammation, DCs have the capacity to capture antigens, migrate to lymph nodes and prime naive T cells, therefore functioning as initiators of T-cell immunity. On the other hand, under steady-state conditions, DCs induce peripheral T-cell tolerance [11]. Substantial evidence indicates that DCs mediate T cell unresponsiveness through induction of tryptophan catabolism mediated by indoleamine 2,3-dioxygenase (IDO) [12]. Depletion of tryptophan has significant effects on T lymphocytes, leading to their anergy and apoptosis [13,14]. Fallarino *et al.* [12] found that induction of IDO depends on the engagement of CD200 with its receptor CD200R1 on the surface of DCs.

CD200 is a type I transmembrane glycoprotein that binds to its receptor CD200R1 on macrophages and dendritic cells, resulting in the regulation of inflammatory immune responses, cytokine production and maintenance of immune homeostasis [15]. In addition, Gorczynski *et al.* [16] demonstrated that the interaction between CD200 and CD200R1 was implicated in the development of tolerogenic DCs that preferentially induce populations of CD4^+^CD25^+^ regulatory T cells (Treg) capable of dampening or preventing immune responses. Treg cells are especially important in the intestine, where the mucosa is exposed to a wide range of foreign antigens, including indigenous flora and dietary antigens [17]. Defects in the number or function of Treg was found to be associated with a breakdown in intestinal tolerance that may contribute to the development of IBD [18].

Blocking the interaction of CD200R1 with its ligand was found to activate DCs and promotes T helper-17 (Th17) differentiation [19]. Recently, it has been found that elevated levels of IL-17 may contribute to the pathogenesis of IBD [20].

CD200/CD200R1 signaling has been suggested to play a role in the induction of autoimmune diseases. In rodents, blocking CD200/CD200R1 binding was found to aggravate the clinical course of experimental autoimmune diseases [21,22]. Consistent with this premise, dysfunction in CD200/CD200R1 signaling has been reported in numerous autoimmune diseases, including Parkinson’s disease [23], Alzheimer’s disease [24], rheumatoid arthritis [25], uveoretinitis [26], lupus [27], autoimmune and inflammatory skin disorders [28] and spontaneous fetal loss [29]. Nevertheless, the expression of CD200/CD200R1 has not been determined in patients with IBD. Therefore, we aimed to investigate the frequencies of DCs expressing CD200 or CD200R1 using flow cytometry and correlate the results with Treg (CD4^+^CD25^+^), Th17 (CD4^+^IL-17^+^) and routine biochemical and serological markers in children with IBD.

## 2. Results

### 2.1. Patient Characteristics

Thirty-seven pediatric patients with IBD were included; 23 of them have CD (nine female and 14 male) and 14 UC (four female/10 male). The age range of patients was 9–15 years (mean age 13.25 ± 2.9 years) (Table 1). IBD patients had a significant weight reduction (*p* < 0.001) compared to control subjects. The mean duration of the disease was more than six months in 50% of UC and in 61% of children with CDs. For the patients with CD, the disease location was confined to the terminal ileum in eight (34.8%), colon in four (17.4%) or ileocolon in 11 (47.8%) patients, and the disease behavior was non-stricturing, non-penetrating in 16 (69.6%), stricturing in four (17.4%) or perforating in three (13.04%) patients accordingly. For the patients with UC, disease was classified according to the Paris classification to ulcerative proctitis and left-sided UC in four (28.6%), extensive to hepatic flexure in two (14.3%) and extensive separately (pancolitis) in eight (57.1%). Disease activity assessed according to the Mayo Clinical Colitis Activity Index was found moderate in five (35.7%) and severe in nine (64.3%) patients. The majority of CD and UC patients had been treated with the combination of aminosalicylate (5-ASA) mesalamine with one of the immunosuppressive drugs prednisolone, azathioprine or infliximab.

**Table 1:** Characteristics of IBD patients and healthy controls included in the study.

Parameter	Ulcerative Colitis (*n* = 14)	Crohn’s Disease (*n* = 23)	Healthy Control (*n* = 14)
Age (years)	13.25 ± 2.94	13.73 ± 3.10	15.21 ± 2.41
Sex (M/F)	4/10	9/14	5/9
Weight (kg)	21.43 ± 1.51	33.79 ± 3.89	47.9 ± 4.73
Height (cm)	114 ± 3.09	136.95 ± 5.07	152.8 ± 7.67
Disease duration (months.)	7 ± 3.16	9 ± 4.01	
**Paris classification**			
Location	Left-sided 4 (28.6%)	Terminal ileum 8 (34.8%)	
	Extensive 2 (14.3%)	Colon 4 (17.4%)	
	Pancolitis 8 (57.1%)	Ileocolon in 11 (47.8%)	
Behavior	N/A	Non-stricturing, non-penetrating 16 (69.6%)	
		Stricturing 4 (17.4%)	
		Perforating 3 (13.04%)	
Mayo score	Mild 0	N/A	
	Moderate 5 (35.7%)		
	Severe 9 (64.3%)		
Rectal bleeding	14 (100%)	13 (56.5%)	
Diarrhea	14 (100%)	19 (82.6%)	
**Therapy**			
Prednisone	12 (85.7%)	23 (100%)	
Mesalamine	14 (100%)	18 (78.3%)	
Azathioprine	12 (85.7%)	23 (100%)	
Sulfasalazine	0	10 (43.5%)	

N/A: not applicable.

### 2.2. Biochemical and Serological Markers

Erythrocyte sedimentation rate (ESR), C-reactive protein (CRP) and alkaline phosphatase (ALP) are known to be good predictors of disease activity in IBD. CD patients showed significantly higher CRP than UC and control subjects (*p* < 0.05, 0.01 respectively). Patients with CD and UC had significantly higher levels of ESR and ALP compared to healthy controls, while no significant differences were detected between the two patient groups. Sera from patients and healthy controls were also tested for anti-Saccharomyces cerevisiae antibodies (ASCA) and perinuclear antineutrophilic cytoplasmic antibody (pANCA), and it was found that none of the control group had these autoantibodies. UC patients had significantly higher pANCA than CD patients did (35.7% *vs*. 17.4%; *p* < 0.01). Both ASCA-IgG and ASCA-IgA were significantly higher in CD than UC patients (Table 2).

**Figure 1:**
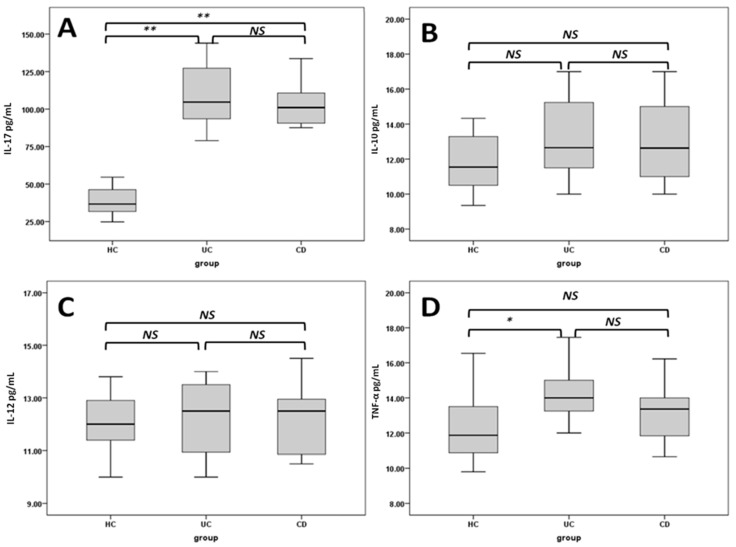
Box plots show the concentrations of IL-17 (**A**), IL-10 (**B**), IL-12 (**C**) and TNF-α cytokines (**D**) in the studied groups. HC: healthy controls; UC: ulcerative colitis patients; CD: Crohn’s disease patients; NS: not significant; * *p* < 0.05; ** *p* < 0.005.

Figure 1 shows that the serum levels of IL-17, IL-10, IL-12 and TNF-α were found to be increased in IBD patients compared to healthy control children. However, significant differences were only found in serum IL-17 of both patient groups (both at *p* < 0.01) and in the TNF-α levels of UC patients (*p* < 0.05) compared to healthy children.

**Table 2:** Biochemical and serological parameters.

Marker	Ulcerative Colitis (*n* = 14)	Crohn’s Disease (*n* = 23)	Healthy Control (*n* = 14)
ALP (IU/L)	175.29 ± 32.24 ^aa,b^	195.15 ± 27.05 ^aa^	67.2 ± 12.74
CRP (mg/dL)	13.57 ± 4.46 ^aa,b^	34.05 ± 8.56 ^aa^	3.42 ± 1.07
ESR (mm/H)	30 ± 4.08 ^a^	35.5 ± 6.23 ^a^	11.2 ± 2.1
pANCA	5 (35.7)% ^bb^	4 (17.4%)	0
ASCA-IgA	2 (14.2%) ^bb^	6 (26.1)%	0
ASCA-IgG	3 (21.4%) ^b^	7 (30.4%)	0

^a^ Denotes statistically-significant difference compared to the control: ^a^: *p* < 0.05, ^aa^: *p* < 0.01; ^b^ Denotes statistical significance compared to Crohn’s disease (CD): ^b^: *p* < 0.05, ^bb^: *p* < 0.01. Values represent the mean ± standard deviations.

### 2.3. Reduced DCs Expressing CD200R1 and Increased CD200^+^DCs

**Figure 2:**
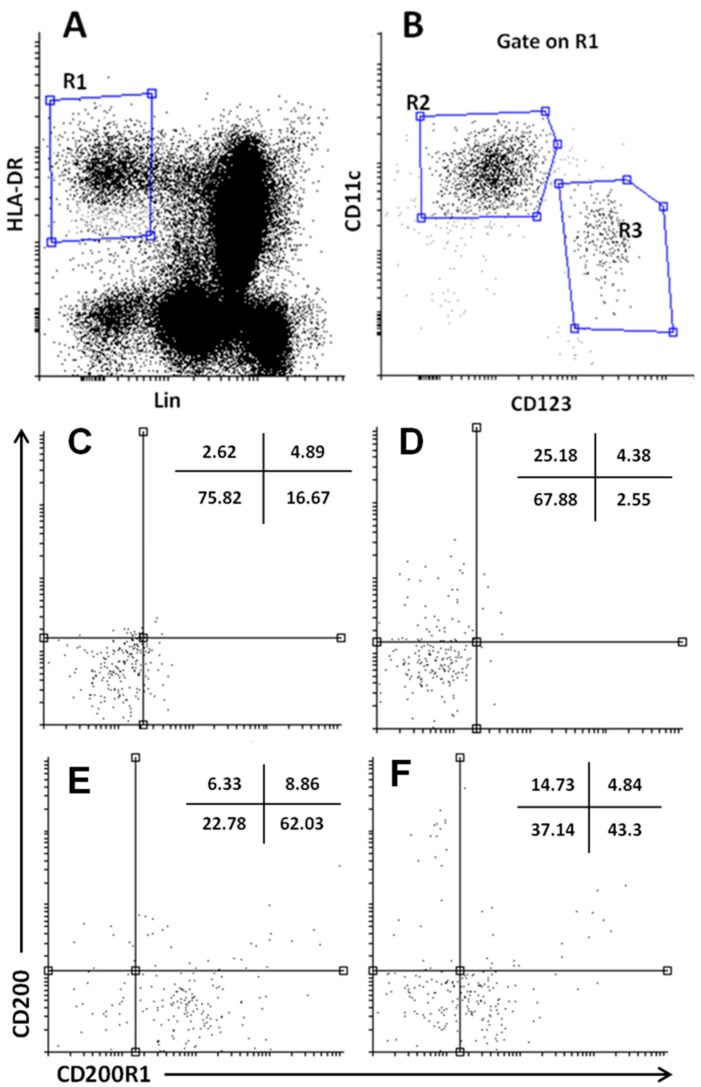
Representative flow cytometry data to illustrate the gating strategy for FACS analysis of DC subsets. (**A**) Dendritic cell HLA-DR^+^Lin^−^ cells were gated (**R1**), and myeloid CD11c^+^ mDCs (**R2**), plasmacytoid CD123^+^ pDCs (**R3**) were determined as a percentage of the total PBMC (**B**). Dot plots to illustrate the expression of CD200 and CD200R1 on the myeloid dendritic cell (mDC) subset of healthy controls and IBD patients (**C**,**D**) and on and plasmacytoid DCs (pDCs) of healthy controls and IBD patients (**E**,**F**).

**Figure 3:**
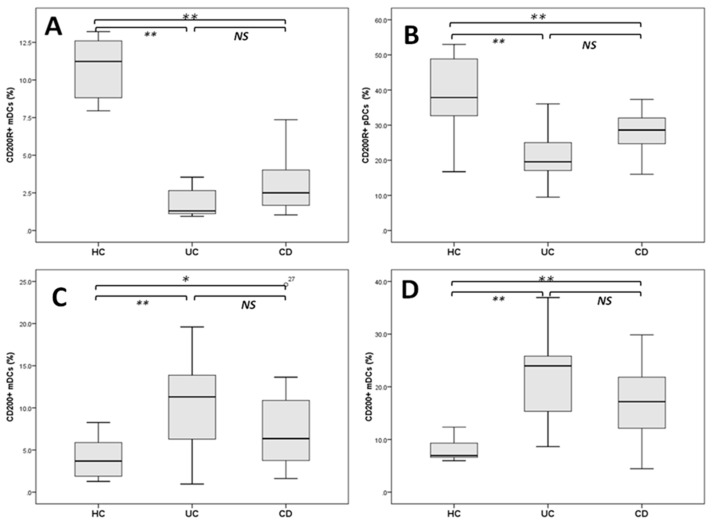
Boxplots show the frequency of CD200R^+^ (**A**,**B**) and CD200^+^ (**C**,**D**) on mDCs and pDCs, respectively. HC: healthy controls; UC: ulcerative colitis patients; CD: Crohn’s disease patients; NS: not significant; * *p* < 0.05; ** *p* < 0.005.

Figure 2 demonstrates a flow cytometric analysis of dendritic cell subsets (myeloid dendritic cells (mDCs) and plasmacytoid DCs (pDCs)) and the frequencies of both DCs expressing CD200 and CD200R1 cell surface proteins. The percentage of mDC and pDCs expressing CD200R1 and CD200 molecules is presented in Figure 3. pDCs expressing CD200R1 in patients with CD and UC were significantly lower than those of healthy controls (HCs) (26.61 ± 5.17, 21.12 ± 5.0 *vs*. 40.24 ± 7.05, both at *p* < 0.005). The expression of CD200R1 on mDCs also was significantly lower in patients with CD and UC compared to HCs (3.1 ± 1.7, 2.49 ± 1.4 *vs.* 10.37 ± 3.3, both at *p* < 0.005). In the meantime, we found that pDCs expressing CD200 were significantly increased in CD and UC patients compared to HCs (17.49.1 ± 4.0, 21.1 ± 4.45 *vs*. 8.7 ± 3.7, both at *p* < 0.005). mDCs expressing CD200 were also found to be significantly increased in CD and UC compared to HCs (7.99 ± 2.4, 10.31 ± 1.6 *vs*. 3.99 ± 1.5, *p* < 0.05 and 0.005, respectively).

### 2.4. Regulatory T Cells and TH17 Cells

**Figure 4:**
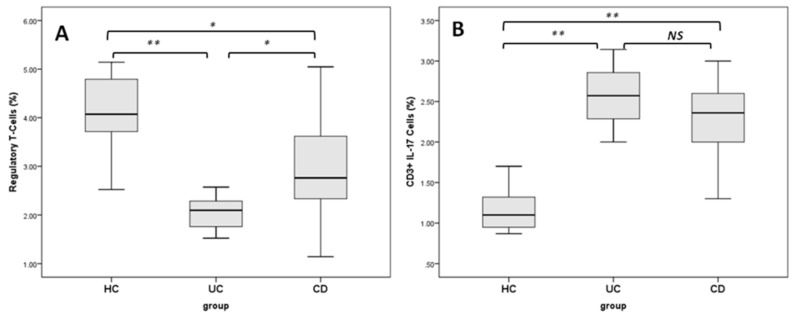
Boxplots show the frequency of Treg (**A**) and Th17 (**B**) among different groups. HC: healthy controls; UC: ulcerative colitis patients; CD: Crohn’s disease patients; NS: not significant; * *p* < 0.05; ** *p* < 0.005.

Regulatory T cells, as identified by flow cytometry based on the expression of CD4, CD25 and Foxp3, were found significantly decreased in UC and CD patients compared to healthy controls (both at *p* < 0.01) (Figure 4A). Th17 cells were determined with intracellular staining of IL-17 in CD4^+^ T cells by flow cytometry, after *in vitro* stimulation by phorbol myristate acetate (PMA)/ionomycin in short-term culture. The percentage of Th17 was found significantly increased in CD compared to UC patients and healthy subjects (2.01% ± 0.28% *vs*. 1.06% ± 0.16% and 0.95% ± 0.14%, *p* < 0.01, 0.001, respectively) (*p* < 0.001). Patients with UC displayed an increased percentage of Th17 cells when compared to that of healthy controls (*p* < 0.001) (Figure 4B).

### 2.5. Correlation Analyses

Correlation analysis showed that none of the biochemical parameters correlated significantly with CD200 or CD200R1, except for ESR and the serum concentration of IL-17, which showed significant negative correlations with DCs^+^CD200R1^+^ (*p* < 0.01, *p* < 0.001, respectively).

Table 3 shows that the percentage of Treg was found significantly correlated with CD200R^+^ mDCs (*r* = 0.383, *p* = 0.023) and CD200R^+^ pDCs (*r* = 0.406, *p* < 0.01). On the contrary, mDCs and pDCs expressing CD200 showed significant negative correlations with Treg (*r* = −0.420, *p* < 0.01; *r* = −0.576, *p* < 0.001, respectively). mDCs expressing CD200R1 were found negatively correlated with Th17 (*r* = −0.633, *p* < 0.0001), whereas CD200^+^ mDCs did not correlate significantly with Th17. pDCs expressing the CD200R1 population were correlated negatively with Th17 CD4^+^ cells (*r* = −0.319, *p* < 0.05); whereas, the CD200^+^ pDC population was found significantly correlated with Th17 (*r* = 0.416, *p* < 0.01) (Table 3).

**Table 3:** Correlations of Treg and Th17 with DC subsets expressing CD200R1 or CD200.

Parameter	Treg	Th17
mDCs	−0.103 (0.568)	0.441 (0.008) **
pDCs	−0.245 (0.169)	0.104 (0.552)
CD200R1^+^ mDCs	0.383 (0.023) *	−0.412 (0.011) *
CD200^+^ mDCs	−0.420 (0.01) **	0.193 (0.251)
CD200R1^+^ pDCs	0.406 (0.012) *	−0.319 (0.001) **
CD200^+^ pDCs	−0.576 (0.001) **	0.416 (0.009) **

** Correlation is significant at the 0.01 level (2-tailed); * correlation is significant at the 0.05 level (2-tailed); values represent the correlation coefficient *r* (*p*-value).

## 3. Discussion

Besides their widely-recognized role as professional antigen-presenting cells in activating T cells, DCs are essential in the regulation of both central and peripheral tolerance. DCs induce tolerance by triggering apoptosis of autoreactive T cells in the thymus (central tolerance) and by their interactions with T cells in the periphery, causing the induction of T cell anergy, T cell apoptosis or the induction of regulatory T cells (Treg) (peripheral tolerance) [30]. DCs possess several costimulatory molecules and immune receptors that enable them to mediate these opposing tasks [31]. CD200R is an inhibitory receptor mainly expressed on myeloid cells that is able to regulate the activation threshold of inflammatory immune responses, both *in vivo* and *in vitro* [32]. CD200, the ligand of CD200R, is a widely-expressed glycoprotein on a wide variety of cells throughout the body. Strong evidence indicates that the interaction between CD200 and CD200R1 is implicated in the development of tolerogenic DCs that preferentially induce populations of CD4^+^CD25^+^ regulatory T cells (Treg) [16] and initiate the immunosuppressive pathway of tryptophan catabolism [12], capable of dampening or preventing immune responses.

In humans, there are two major subsets of circulating DCs that have been identified, termed myeloid (mDCs) and plasmacytoid DCs (pDCs) [33]. pDCs play also a vital role in the induction of oral tolerance, since they prevent oral T cell priming and are responsible for systemic tolerance to dietary antigens, including proteins and haptens [34]. pDCs were further shown to complement the role of Treg cells in inducing oral tolerance initiated in the gut [35]. In contrast, mDCs are highly effective at antigen presentation and T cell stimulation and have the capacity to produce Th1 and induce Th17 responses [36]. In agreement with these data, we found that mDCs correlate significantly with Th-17.

In our study, the percentage of pDCs expressing the tolerance molecule CD200R in healthy controls was higher (40% and 50% of whole pDCs) than that of mDCs (around 10% of whole blood mDCs), suggesting that pDCs are more tolerogenic than mDCs. These observations are in line with previous preclinical and clinical trials suggesting that pDCs exert a stronger immune suppression compared to mDCs and preferentially induce the differentiation of allogeneic Treg [37,38] and, thus, are generally defined as tolerogenic dendritic cells.

In patient groups, our data demonstrate a significant decrease in tolerogenic pDCs expressing CD200R. This finding may provide an explanation for the observed exaggerated inflammatory response of circulating mDCs to lipopolysaccharide (LPS) in patients with inflammatory bowel disease that was reported earlier by Baumgart *et al.* [39]. Furthermore, the percentage of CD200R^+^ DCs was found to be significantly correlated positively with CD4^+^CD25^+^ Treg and to have a significant negative correlation with TH17 cells. These data are in agreement with Ochando *et al.* [40], who reported that pDCs can induce the generation of CD4^+^CD25^+^Foxp3^+^ Treg cells and that depletion of pDCs or prevention of pDC lymph node homing inhibited peripheral Treg cell development and tolerance induction.

Recently, it has been demonstrated that Th17 and Treg cells have a functional antagonism, in which Tregs act as immunosuppressive cells, and Th17 cells are involved in initiating autoimmune and inflammatory diseases [41]. The balance between Treg and Th17 cells is tightly regulated. Dysregulation of this balance can lead to inflammation and autoimmunity. Interestingly, we found that the reduction in CD200R+ DCs is associated with increased TH17, and decreased Treg cells may give a clue to elucidate the cause of TH17/Treg imbalance, which is possibly the leading cause of IBD, as suggested by Eastaff-Leung *et al.* [42].

On the contrary, we found that IBD patients have a significant increase in DCs expressing CD200, both in UC and CD patients. In addition, we found a significant positive correlation between CD200^+^pDCs and Th17. CD200 has one major difference from CD200R in that it does not contain signaling motifs or docking domains in its short cytoplasmic tail; therefore, no direct effect was observed on cells expressing it upon its binding to CD200R [43]. Actually, several studies have indicated that CD200 imparts a unidirectional negative signal on binding to CD200R-bearing cells [32,44]. Mukhopadhyay *et al.* [45] demonstrated that induction of CD200 limits cell activation and protects the host from excessive inflammation. In addition, upregulation of CD200 expression was found to be associated with diminished proinflammatory cytokine production to self-antigens [46]. Based on these data and our results, it can be suggested that the increase in DCs expressing C200 may be considered as a compensatory mechanism to downregulate their exaggerated inflammatory immune response, as previously suggested by Jurgens *et al.* [47].

## 4. Experimental Section

### 4.1. Study Population

The study included 37 consecutive unselected cases with IBD that were referred to the outpatient Pediatrics Clinic of King Abdulaziz University Hospital (KAUH, Jeddah, Saudi Arabia) during the period from May 2013 to May 2014. Demographic and clinical data were collected from all patients, including gender, ethnicity, age, age at diagnosis, disease activity, their history of surgery related to IBD, chronic steroid use (including steroid-dependent or steroid-refractory disease) and the presence of side effects of medical treatment. The disease activity was assessed according to the Paris modification of the Montreal classification for IBD [48]. The clinical activity of UC patients was evaluated according to the Mayo score [49]. Patients were excluded if they had incomplete ileocolonoscopy, microscopic colitis, infectious ileocolitis, colorectal cancer, colorectal polyps, unclear diagnosis, urinary incontinence, no fecal samples, viral infection (HIV, hepatitis B or C), chronic steroid use (including steroid-dependent or steroid-refractory disease) and the presence of side effects of medical treatment. Fourteen age-matched pediatric volunteers (5 males and 9 females), who were clinically and laboratory free of any autoimmune diseases, served as a healthy control group. For understandable reasons, subjects in the healthy control group had no endoscopic workup. The study protocol was approved by the Ethics Committee of the KAUH, and the study has been conducted according to the principles expressed in the Declaration of Helsinki. Patients or one of their parents were informed of the nature of the investigation and were instructed to complete the questionnaire. The clinical characteristics of the patients are shown in Table 1.

### 4.2. Sample Preparations

Ten milliliters of blood were drawn from each participant. Each blood sample was divided into two equal parts; 5 mL of blood were collected in serum separator tubes, centrifuged and assayed immediately for routine biochemistry tests. The other part of the blood sample was collected into tubes with anticoagulant ethylenediaminetetraacetic acid (EDTA) for isolation of PBMCs from whole blood using Ficoll-Paque (GE Health Care, Boston, PA, USA) density gradient centrifugation. Cells were washed once with RPMI-1640 (BioWhittaker, Walkersville, MD, USA) and prepared for flow cytometry assays.

### 4.3. Biochemical and Serological Investigations

The serum concentrations of C-reactive protein (CRP), erythrocyte sedimentation rate (ESR), ALB (albumin), ALT (alanine aminotransferase), AST (aspartate aminotransferase), ALP (alkaline phosphatase) and GGT (glutamyl transferase) were analyzed using commercially available kits (Dade*-*Behring*,* Marburg, Germany) according to the manufacturer’s instructions. The levels of IgG and IgA antibodies to ASCA were detected in serum using ELISA kits (Inova Diagnostics Inc., San Digo, CA, USA) that were performed in duplicate according to the manufacturer’s instructions. pANCA was analyzed by ELISA and indirect immunofluorescence, as previously described [50]. The concentrations of TNF-α, IL-17, IL-12p40 and IL-10 were determined in the sera of all of the participants by the enzyme linked immunosorbent assay (ELISA) using the Quantikine kits (R&D Systems, Minneapolis, MN, USA). The minimum detectable levels using the ELISA kits were 5.5 pg/mL for TNF-α, 15 pg/mL for IL-17, 5 pg/mL for IL-12, 3.9 pg/mL for IL-10. The tests were conducted according to the manufacturer’s instructions.

### 4.4. Flow Cytometry Assays

#### 4.4.1. Dendritic Cells Subsets Analysis

Plasmacytoid and myeloid subsets of DCs from PBMCs were identified by multiparametric flow cytometry as described elsewhere [51]. Briefly, 100 µL of PBMCs were stained with FITC-lineage cocktail 1 (consists of anti-CD3, -CD14, -CD16, -CD19, -CD20 and -CD56) (Becton Dickinson, San Jose, CA, USA), PE-CD200R1 (Clone 380525, R&D Systems), PerCP-CD123 (Clone 32703, R&D systems), APC-CD200 (Clone 380525, R&D Systems), AF700-CD11c (Clone ICRF 3.9, R&D systems) and PE-Cy7-HLA-DR (Clone L243, BD-biosciences, San Jose, CA, USA). Isotype-matched control mAbs were used to determine the nonspecific binding. Flow cytometry analysis was performed on a Navios flow cytometer (Beckman-Coulter Inc., Brea, CA, USA). Fifty thousand events were acquired, and data were analyzed with the Summit v4.3 Build 2445 software (Dako-cytomation Inc., Carpinteria, CA, USA). Cells were gated by their forward- and side-scatter properties and identified further by specific surface markers. The lineage-negative cells were analyzed for the expression of CD11c, CD123 and human leukocyte antigen (HLA)-DR. Plasmacytoid DCs (pDCs) were identified as cells double-positive for CD123 and HLA-DR, while myeloid DCs (mDCs) were identified as cells double positive for CD11c and HLA-DR (Figure 1A). Percentages of CD200 and CD200R1 were determined after gating on mDCs and pDCs (Figure 1C,D).

#### 4.4.2. Regulatory T Cells Analysis

For regulatory T cell staining, 1 × 10^6^ cells were resuspended in 100 μL flow cytometry staining buffer (R&D Systems, Minneapolis, MN, USA). Cells were incubated with FITC-labelled anti-CD4 (clone FAB3791F, R&D Systems) and APC-labelled anti-CD25 (clone BC96, R&D Systems) antibodies for 30 min at 4 °C in the dark. For intracellular staining, after permeabilization with fixation/permeabilization buffer (R&D Systems), phycoerythrin *(*PE*)*-labelled anti-Foxp3 antibody (clone IC8214P, R&D Systems) was added and incubated for 30 min at 4 °C in the dark. FITC- and APC-conjugated mouse IgG2a and PE-conjugated rabbit IgG antibodies were used as the isotype control antibodies.

#### 4.4.3. Th17 Cells’ Analysis

PBMCs were cultured in RPMI-1640 complete medium supplemented with 10% (*v*/*v*) FBS, 2.0 mM l-glutamine, 50 mM 2-mercaptoethanol, 100 U/mL penicillin and 100 mg/mL streptomycin. Cells were activated with 50 ng/mL phorbol myristate acetate (PMA) plus 1.0 μg/mL of ionomycin for 6 h in the presence of 0.5 μg/mL brefeldin-A (all from Sigma-Aldrich, St. Louis, MO, USA); then, cells were fixed for 10 min at room temperature with PBS containing 4% paraformaldehyde (Sigma-Aldrich). The control PBMCs were cultured in medium alone. The stimulated PBMCs were harvested and stained with FITC-labeled anti-CD4. After 30 min of incubation, cells were fixed with the Perm/Fix solution, and permeabilized, followed by staining with PerCP-labeled anti-IL-17 (Clone 41802, R&D Systems). Ten thousand events were acquired by flow cytometry, and the percentages of cells producing IL-17 cytokine were determined after gating on CD4^+^ lymphocytes. Appropriate conjugated IgG antibodies were used as isotype controls. All flow cytometry data were acquired on a Beckman-Coulter Navios cytometer (Beckman-Coulter Inc., Brea, CA, USA) and analyzed with Summit software (Dako-cytomation Inc., Carpinteria, CA, USA).

### 4.5. Statistical Analysis

All data were statistically analyzed using the Statistical Package for Social Sciences (SPSS) V20.0. The data were expressed as the means ± standard deviation (SD). Correlations were done using the Pearson tests, and analyses of variance were done using the ANOVA test followed by Bonferroni correction. A *p*-value less than or equal to 0.05 is considered as being statistically significant.

## 5. Conclusions

In summary, our study demonstrates that IBD is characterized by a decreased prevalence of mDCs and pDCs expressing the inhibitory molecule CD200R. Furthermore, our data show that CD200R1 expression on DCs correlates positively with circulating Treg and negatively with ESR and Th17. Finally, our findings suggest that DCs are implicated in the pathogenesis of IBD and that they exert their effects, at least in part, through modulation of Th17/Treg balance. Further functional studies, as well as expanding investigation on other cells and mediators of immune responses and inflammation are currently in progress by our research group to better elucidate the molecular mechanism of IBD and to provide a basis for identifying novel therapeutic targets in those autoimmune disorders.
